# Effect of *Oudemansiella raphanipies* Powder on Physicochemical and Textural Properties, Water Distribution and Protein Conformation of Lower-Fat Pork Meat Batter

**DOI:** 10.3390/foods11172623

**Published:** 2022-08-29

**Authors:** Yingying Zhao, Yanqiu Wang, Ke Li, Igor Mazurenko

**Affiliations:** 1 College of Food and Bioengineering, Zhengzhou University of Light Industry, Zhengzhou 450001, China; 2 Department of Food Technology, Sumy National Agrarian University, 40021 Sumy, Ukraine; 3 School of Agriculture and Biotechnology, Hunan University of Humanities, Science and Technology (HUHST), Loudi 417000, China

**Keywords:** *Oudemansiella raphanipies*, emulsified meat products, cooking yield, rheology, LF-NMR, Roman spectroscopy

## Abstract

The effects of the addition of different amounts (0%, 1%, 2%, 3% and 4%) of *Oudemansiella raphanipies* powder (ORP) to lower-fat pork batter on its physicochemical, textural and rheological properties, water distribution and protein conformation were evaluated. The results showed that the addition of ORP from 0% to 4% significantly decreased the pH and L* value of pork batter (*p* < 0.05); however, it also increased the a* value and enhanced the cooking yield of pork batter from 77% to 92%. Pork batter with 1–2% ORP added had an improved texture profile and a higher storage modulus (G’), but the addition of 3–4% ORP resulted in an inferior texture of pork batter and G’. LF-NMR showed that the addition of ORP significantly increased the peak area ratio of immobile water and reduced the peak area ratio of free water (*p* < 0.05). ORP significantly affected protein secondary structure of pork batter. The α-helix content of pork batter with 1–2% ORP decreased and β-sheet content increased. Overall, the addition level of 1–2% ORP effectively improved the texture and water holding capacity of lower-fat emulsified sausage and provides a new reference for developing nutritional meat products.

## 1. Introduction

Emulsified meat products, such as sausages, meatballs and burgers, are the most popular ready-to-eat foods for consumers because of their convenience and high sensory quality [1]. In general, emulsified meat products are produced with a high level of fat, ranging from 20% to 30% [2]. Excessive consumption can easily lead to obesity and certain chronic diseases, threatening human health. As consumers become increasingly aware of the relationship between red meat consumption and health, the decrease in the fat content of emulsified meat products has been one of the developing directions of the meat processing industry. Animal fat plays a key role in determining the quality of emulsified meat products, such as improved texture, cooking yield, color and unique flavor [3]. Previous studies have reported that the direct fat-reduction method by the addition of more water or meat leads to greater cooking loss and inferior emulsion stability of final products [4]. It remains a challenge to reduce the content of animal fat and improve or maintain the quality of lower-fat meat products. Meat scientists and producers introduce bioactive components during processing and design novel formulations to increase product value of processed meat, thus achieving a balance between fat replacement and desirable meat quality [5,6]. In recent years, edible fungi have been added into meat emulsions systems to obtain healthier meat products with lower fat, reduced salt and fewer calories, as well as the introduction of bioactive components, such as dietary fibers and natural antioxidants.

Edible fungi are rich in unsaturated fatty acids, amino acids, minerals and other nutrients, as well as bioactive substances such as polysaccharides and phenols, which represent a much healthier alternative for a balanced diet [7,8]. These nutrients have antioxidant, bacteriostatic and anticancer functions. Edible fungi are widely used in meat product processing and are advantageous in improving the nutritional attributes, textural properties and cooking yield of finished meat products [9,10]. Recent studies concerned the addition of various edible mushroom flours into meat products for decreasing salt and fat content and improving the quality of the final products, such as water retention, textural characteristics and flavor [11,12,13]. Cerón-Guevara et al. found that the addition of *Agaricus bisporus* and *Pleurotus ostreatus* flours as a partial substitute of fat and salt into cold-stored beef patties effectively improved its cooked yield, but did not improve the springiness and cohesiveness compared to the high-fat beef patties [14]. Patinho et al. reported that the fat could be partially replaced by the addition of 10% and 20% *Agaricus bisporus* in beef burgers to produce low-fat beef burgers, while retaining the instrumental characteristics and a good overall acceptability [15].

*Oudemansiella raphanipies* is a rare edible fungus with fresh and tender meat, unique taste and high nutritional value, which has been welcomed by consumers [7]. In China, it is mainly distributed in Yunnan, Sichuan, Fujian, Xizang, Jiangsu and other places. *Oudemansiella raphanipies* contains high protein, low fat and a high content of umami amino acids and antioxidant components such as polyphenols, polysaccharides and flavonoids. At present, more research has focused on the activity characterization of functional components in the *Oudemansiella raphanipies*. Thus, it has potential as a functional food used in analgesic, anti-inflammatory contexts, improving immunity, repairing damaged organs and regulating body functions, which may be applied in meat products to improve nutritional values and functional properties. However, the application of *Oudemansiella raphanipies* powder (ORP) in the development of meat products is scarce. It is essential to further understand whether ORP has the ability to promote the formation of meat proteins matrix formation and a better water-holding capacity (WHC) of lower-fat meat batter.

Therefore, the aim of this work was to investigate the effect of ORP on the physicochemical, textural and rheological properties, water distribution and movement and protein secondary structure of lower-fat pork batters. Our findings will provide a promising method to achieve nutritious meat products, while maintaining texture and water retention.

## 2. Materials and Methods

### 2.1. Materials

*Oudemansiella raphanipies* was obtained from Yichun Minghe Kangtai Agricultural Development Co., Ltd. (Nanchang, Jiangxi, China). Pork leg lean meat (M. *semitendinosus*, 24–48 h postmortem, pH 5.6–5.8), pork back fat, salt, sugar and white pepper powder were purchased from Zhengzhou Dennis Department Store Co., Ltd. (Zhengzhou, Henan, China). All other chemicals and reagents used in this study were of analytical grade.

### 2.2. Preparation of Oudemansiella raphanipies Powder

The *Oudemansiella raphanipies* fungi were washed in warm water at 60 °C to remove sediment, and dispersed into triple distilled water (w:w) with an ultrasound cleaner (Ningbo Xinzhi Biotechnology Co., Ltd., Ningbo, China) at 300 W for 30 min. The samples were dried at 80 °C for 16 h (electric constant humidity air drying box, DHG-9076A Shanghai Jinghong Experimental Equipment Co., Ltd., Shanghai, China). Next, they were grounded to a powder by high-speed mill (QE-100, Zhejiang Yili Industry & Trade Co., Ltd., Quzhou, China). Afterward, they were sieved by 60 mesh sieve (aperture 0.28 mm) to obtain *Oudemansiella raphanipies* powder (ORP). The ORP was repacked in a vacuum bag and stored at room temperature for subsequent experiments.

### 2.3. Determination of Chemical Composition and Hydrolytic Amino Acid Content of OPR

The moisture, protein, crude fat, carbohydrate, crude fiber and ash contents of *Oudemansiella raphanipies* powder were measured according to the method of Yu et al. (2020) [16]. Amino acid composition of ORP was determined according to the guideline of GB 5009.124-2016 [17].

### 2.4. Preparation of Lower-Fat Meat Batters

Five groups of lower-fat pork meat batters were formulated, as presented in Table 1. Specifically, the C group was set as the control. The other four treatment groups, labeled T1, T2, T3 and T4, were formulated with 1%, 2%, 3% and 4% (*w*/*w*) ORP of the meat (M_meat_ = M_Pork lean meat_ + M_Pork back-fat_; M stands for mass), respectively. Details for meat batter preparation were described in the previous study [18]. Briefly, all external fat and connective tissue were removed from the meat. The pork lean meat and pork back-fat were crushed by a 6 mm meat grinder (HM 740, Qingdao Hanshang Electric Appliance Co., Ltd., Qingdao, Shandong, China) and then frozen for later use.

The abovementioned minced meat was thawed at 4 °C overnight. A total of 160 g pork leg lean meat was ground with the required amount of *Oudemansiella raphanipies* powder and 10 g ice water in a bowl chopper (Retsch GM 300, Haan, Germany) at 3000 rpm for 20 s. Next, 3 g salt, 0.48 g tripolyphosphate and 10 g ice water were added into meat batter and chopped for another 20 s, followed by a 2 min break. Afterward, 40 g pork fat, 7.2 g sugar water, 0.32 g white pepper and 10 g ice water were added to the mixtures and chopped for 20 s. The prepared raw meat batter was stored at 4 °C for later use.

Approximately 20 g of the prepared meat batter was stuffed into 50 mL polypropylene centrifuge tube and sealed hermetically. To remove the residual air bubbles, the plastic containers were centrifuged at 500× *g* for 5 min at 4 °C. Some of the centrifuged meat batters were heated in a water bath at 80 °C for 20 min until the core temperature reached 72 °C, then chilled immediately in an ice bath for 30 min and kept overnight at 4 °C for the recording pH measurement, color, texture profile analysis (TPA) and Raman spectroscopy. Each group was made in six parallel samples. A total of thirty meat batter samples were obtained (6 × 5 groups). The remaining meat batters were stored at 4 °C to evaluate the dynamic rheology, cooking yield, water retention measurement and low-field nuclear magnetic resonance (LF-NMR) spectroscopy.

### 2.5. pH

After cooling at 4 °C overnight, the cooked lower-fat pork batters were placed at room temperature (20 °C) for 2 h. Approximately 10 g of the cooked meat batter was blended with 40 mL distilled ice water using a homogenizer at 15,000 rpm for 10 s (T25 digital, IKA Ltd., Staufen, Germany), and the pH was measured by a digital pH meter (Hanna, Italy). Each treatment group was performed in three replicates.

### 2.6. Color Evaluation

The color of cooked lower-fat pork batter was determined according to the method of Li et al. [18] with some modifications. After cooling at 4 °C overnight, the cooked lower-fat pork batters were placed at room temperature (20 °C) for 2 h. Then, the boiled pellets were cut into 2 cm-thick cylinders, and then the color of the samples was measured with a portable high-quality spectrophotometer (NS 800, 3 NH Technology Co., Ltd., Shenzhen, China) calibrated with an 8 mm aperture and D_65_ illuminant.

Before testing, the instrument was immediately calibrated against a standard white plate (L* = 96.28, a* = 0.26, b* = 1.26). The lightness (L*), redness (a*) and yellowness (b*) values were recorded individually. Six samples taken from each formulation group were evaluated for the internal color.

### 2.7. Cooking Yield

The cooking yield (%) of the samples was measured and evaluated based on the procedure described by Sompugdee et al. [5]. Briefly, after refrigerated storage at 4 °C overnight, the meat batters prepared in Section 2.4 were kept at room temperature for 30 min to achieve an equilibrium. We poured the sample out of the centrifugal tube and removed the excess water with absorbable paper and weighed the mass meter (m_3_) of each pellet sample.

The cooking loss (%) was calculated as a percentage from the known weights of meat batter before cooking.

Cooking yield/% = m_3_/(m_2_ − m_1_) × 100%

where m_1_ is the mass of an empty 50 mL centrifugal tube (g); m_2_ is the total mass of sample and centrifugal tube before cooking (g); and m_3_ is the sample mass (g) of dried water after cooking.

### 2.8. WHC

Approximately 1.00 g (exact sample weights were recorded) of meat batter was wrapped in a double filter paper and filled into 50 mL plastic tubes, centrifuged at 3500 rpm for 5 min at 4 °C. After centrifugation, we removed and dried the surface moisture and measured its mass immediately. The pre-centrifugation weight (m_4_) and the post- centrifugation weight (m_5_) of the sample were accurately weighed to be used for the following calculation formula:

WHC/% = m_5_/m_4_ × 100% (1)

### 2.9. Texture Profile Analysis

The cooked meat batters prepared in Section 2.4 were equilibrated at 25 °C for at least 2 h to keep the internal and external temperature of the pellet consistent, and then cut into a 20 mm-high meat cylinder. Textural profile analysis (TPA) was conducted using a texture analyzer (Model TA-XT Icon, Stable Micro Systems Co., Ltd. Godalming, Surrey, UK) equipped with a P/36R cylindrical probe. The setting parameters were as follows: pre-test speed, 2.0 mm/s; test speed, 2.0 mm/s; post-test speed, 5.0 mm/s; strain, 50%; time, 5 s; trigger type, automatic. The obtained parameters comprise hardness, springiness, cohesiveness and chewiness. Hardness is the peak force for the first compression (g). Springiness is the ratio of the sample recovered after the first compression (mm). Cohesiveness is the ratio of the area of the second compression to the first compression (dimensionless). Chewiness is the product of hardness × springiness × cohesiveness (g.mm). Each parallel treatment group was tested six times.

### 2.10. Dynamic Rheological Measurement

To measure the dynamic rheological behaviors of the uncooked lower-fat meat batter during thermal processing, a temperature ramp sweep test of samples was performed using a hybrid rheometer (Discovery HR-1, TA Instruments Inc., New Castle, DE, USA) following the method of Li et al. [19]. Approximately 5 g of uncooked low-fat meat batter was placed between the 40 mm diameter smart-swap, the gap between upper and lower of the parallel plates was 0.5 mm in diameter, and then the edge of sample was sealed with a thin layer of silicone oil to avoid moisture evaporation during the thermal processing. The dynamic viscoelastic measurements were determined at a 0.5% strain and a fixed frequency of 0.1 Hz in an oscillating mode. The heating procedure was programmed to hold each sample for 10 min at 20 °C, followed by a heat-induced process from 20 °C to 80 °C at a heating rate of 1 °C/min via a programmable circulating water bath. The rheological storage modulus (G′) and loss modulus (G″) were recorded continuously during the gel-forming process. Each sample was performed in triplicate.

### 2.11. Low-Field NMR Measurement

Approximately 1.0 g of raw meat batter was put into a 1.5 mL centrifuge tube at 80 °C for 20 min, then further cooled at room temperature for 2 h. The meat sample was taken out and the surface moisture was dried. The meat sample was put into a new 1.5 mL centrifuge tube and then put into a nuclear magnetic tube with a diameter of 15 mm. Low-field NMR was determined in the analyzer, which was operated at a proton resonance frequency of 18 MHz at 32 °C. Spin–spin relaxation time T_2_ was measured with a Carr-Purcell-Meiboom-Gill (CPMG) sequence, resulting in an exponential decay pattern. The operation conditions were as follows: the sampling frequency was 200 kHz and the half-echo time τ- (the time between pulse of 90° and 180°) was 200 µs. Data from 12,000 echoes were acquired as 16 scan repetitions with the interval of 110 ms. Data of six measurements per samples were recorded.

### 2.12. Raman Spectrometry Analysis

The Raman spectra of cooked pork batter were measured using a Laser Confocal Raman Microscope (inVia Qontor, Renishaw, UK) equipped with a 532 nm argon ion laser. Approximately 0.5 g of sample was put into the center of a slide, and a Raman spectrum in the range of 400~3600 cm^−1^ was collected. The test parameters of each spectrum were acquired as follows: 3 scans, 30 s of exposure time, 1 cm^−1^ resolution and laser power was 10% of the total power. The obtained spectra for baseline and smooth were analyzed using Peakfit 4.12 software. The secondary structures including the α-helix, β-sheet, β-turn and random coil content of samples were determined based on the method of Alix et al. [20].

### 2.13. Statistical Analysis

Statistical analysis was performed by one-way ANOVA using SPSS V.21.0 (SPSS Inc., Chicago, IL, USA). A significant difference among lower-fat pork batter samples with or without ORP was determined at the *p* < 0.05 level and verified by Duncan’s multiple range test. The results are displayed as the mean ± standard deviation (Means ± SD). The software Origin 8.5 was used to make the figures.

## 3. Results and Discussion

### 3.1. Analysis of Chemical Constituents of ORP

The chemical composition of ORP is shown in Table 2. Protein accounted for a higher proportion (26.92%). The nutritional value of mushrooms is mainly related to a protein content. Mushroom protein is considered to have higher nutritional quality compared to plant proteins [21].

Table 3 shows the composition and content of hydrolyzed amino acids in *Oudemansiella raphanipies* powder, which contains 17 kinds of amino acids, including 7 kinds of essential amino acids, accounting for 50.83% of the total hydrolyzed amino acids. It is a high-quality protein source. Phenylalanine is an essential amino acid for humans, which means that it cannot be synthesized and must be obtained from food. As shown in Table 3, the content of phenylalanine is the largest, accounting for 16.68% of the total amino acid composition. Aspartic acid and glutamic acid are known as umami amino acids, which are similar to monosodium glutamate and can provide mushrooms with an appealing umami flavor. These two amino acids account for 18.13% of the total hydrolyzed amino acids of ORP and are one of the main sources of aroma components of *Oudemansiella raphanipies* [22]. It was also reported that four new brain glycosides promoting nerve protrusion were found in *Oudemansiella raphanipies*. In addition, various active ingredients such as saponins, coumarins, ergosterol and polyphenols were also found in *Oudemansiella raphanipies* [23,24,25].

### 3.2. pH and Color of Cooked Lower-Fat Meat Batter

The pH and color changes of the cooked pork batters are shown in Table 4. With the increase in the amount of ORP added to the meat batter, the pH value showed a significant downward trend. The pH value of the control group was 6.04, and when the amount of ORP added was 6 g, the pH value decreased most, up to 5.97. However, Patinho et al. (2021) [15] reported that the addition of *Agaricus bisporus* mushroom to beef burgers did not cause significant changes in the pH value. These differences may be partially attributed to the chemical composition of mushroom or formulation of meat batters. It is speculated that the decrease in pH value may be due to the high content of acid amino acids in ORP (18.13%). The results of Choe et al. showed that when mushroom powder was added into sausage, the pH of the sausage increased due to the high content of alkaline amino acids in mushroom powder [26].

The color changes of the cooked pork batter are shown in Table 4. Overall, the lightness value (L*) decreased with the increase in the addition level of ORP added, and the L* value in the control group was 75.33. When the addition level of ORP was 4%, the L* value decreased to 58.27. On one hand, it was speculated that the color change of the meat batter was related to the color of ORP. Li et al. [18] reported that the decreased L* value was related to the color of added material and its content when added to meat batters. On the other hand, L* value of the meat patties was related with the WHC. The higher cooking yield of pork batters may contribute to the smaller L* value. Kurt and Gençcelep (2018) [13] found that a higher amount of *Agaricus bisporus* mushroom powder added to the meat emulsion reduced the L* value. Redness value (a*) increased from 0.07 to 3.39 with the increase in ORP. Yellowness value (b*) had no significant change (*p* > 0.05). Similar findings were reported by Patinho et al. (2021) [15] in beef burger containing *Agaricus bisporus* mushroom. They indicated that the added mushroom inhibited the lipid oxidation and kept the oxymyoglobin stable to prevent its transition to metmyoglobin, thus the addition of mushroom power increased the a* values.

### 3.3. Cooking Yield and WHC

**Figure 1:**
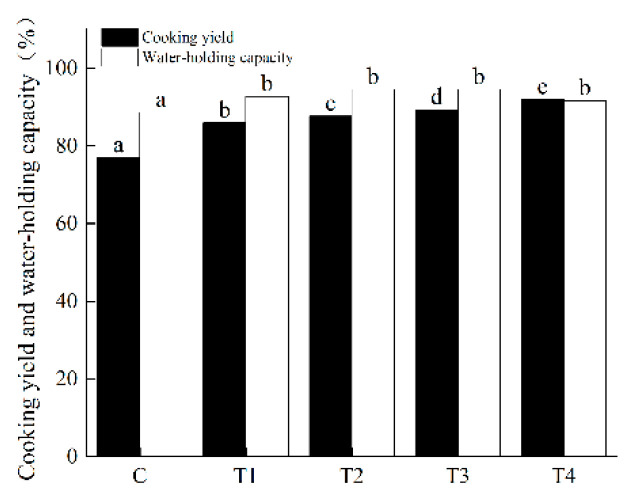
Cooking yield and water-holding capacity of cooked pork batters prepared with various amounts of ORP; C: meat batter without ORP; T1: meat batter containing 1% ORP; T2: meat batter containing 2% ORP; T3: meat batter containing 3% ORP; T4: meat batter containing 4% ORP; different lowercase letters from black column (a, b, c, d, e) indicate significant differences (*p* < 0.05) in cooking yield; different lowercase letters from white column (a, b) indicate significant differences (*p* < 0.05) in WHC.

The cooking yield and WHC of pork batters were significantly influenced (*p* < 0.05) by the amount of added ORP (Figure 1). The control pork batter had the lowest cooking yield. As the content of ORP added increased from 1% to 4%, the cooking yield significantly increased (*p* < 0.05), which indicated that the addition of ORP could enhance the water- and fat-holding capacity of pork batters. Similar results have been reported by Patinho et al., who found that as the concentration of *Agaricus bisporus* mushroom addition increased, the cooking loss of beef burgers decreased [15]. These results were highly related to the higher content of dietary fiber with the increased addition of ORP [27]. In general, the dietary fiber exhibited a higher water- and fat-holding capacity to reduce water loss during cooking [18]. Regarding WHC, it was observed that the cooked pork batters with the addition of ORP showed significantly enhanced WHC (*p* < 0.05). Interestingly, although the WHC of the cooked pork batters presented a change between treatments, it was not significantly different (*p* > 0.05) among T1, T2, T3 and T4. This indicated that added ORP in pork batter formulation effectively improved the water retention of meat products.

### 3.4. Texture Profile Analysis and Appearance

The effect of various amounts of ORP on the textural properties of lower-fat pork batters is shown in Table 5. The addition of ORP has a significant effect on the hardness, springiness and chewiness (*p* < 0.05). Compared to the control, the hardness and springiness significantly increased (*p* < 0.05) when ORP added into meat batter formulation increased from 0% to 2%, The 2% ORP preparation was the highest. However, the addition of either 3% (T3) or 4% (T4) ORP in pork batter formulation decreased the hardness. The springiness was not significantly different (*p* > 0.05) among C, T3 and T4. This may be because the appropriate addition of ORP acted as “active” binders, thus promoting the interaction of proteins and enhancing the formation of a better gel network [28]. The higher content of incorporation ORP into meat batter disturbed the protein gelling fiber [29]. Another reason may be that the cooked batter formulated with the increased addition of ORP had higher water content and less cooking loss. Gao et al. reported that the hardness varies inversely with the water retention of the ground pork patties when adding a higher content of glutinous rice flour [30].

The cohesiveness was not significantly different (*p* < 0.05) between the control and 1% ORP addition. However, the increased addition of ORP from 2% to 4% resulted in a greater reduction in the cohesiveness of cooked pork batters. Cerón-Guevara et al. found that a higher content of added *Agaricus bisporus* and *Pleurotus ostreatus* flour reduced the cohesiveness of frankfurter sausages [31].

The chewiness significantly increased (*p* < 0.05) when adding 1% ORP into the raw pork batters. Then, the chewiness of cooked lower-fat pork batters decreased significantly (*p* < 0.05) when the addition of ORP was more than 2%. Similar results were reported by Syuhairah et al. and Patinho et al., who found that adding higher vegetable or mushroom incorporation lowered the chewiness of sausages and beef burgers [15,32].

The chewiness is obtained by the result of hardness × cohesiveness × springiness. The addition of ORP had a greater influence on the hardness among these three parameters (Table 5). As a result, the cooked batter with the higher content of ORP addition required less energy to be chewed, which was attributed to the samples formulated with more added ORP proportion becoming softer [15]. Overall, the changes in TPA attributes (Table 5) indicated that lower-fat pork batters with ORP content ranging from 1% to 2% improved or maintained the textural properties of the cooked meat batters.

**Figure 2:**
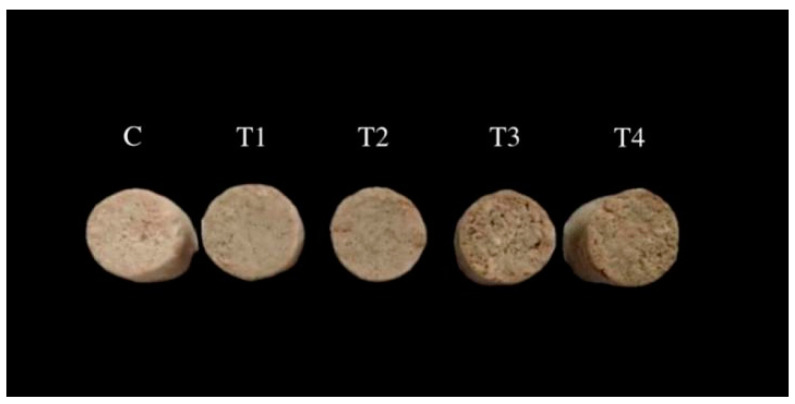
Transverse sections of cooked lower-fat pork batters prepared with various amounts of ORP; C: meat batter without ORP; T1: meat batter containing 1% ORP; T2: meat batter containing 2% ORP; T3: meat batter containing 3% ORP; T4: meat batter containing 4% ORP.

Figure 2 shows the appearance of cooked lower-fat pork batters with various levels of ORP addition. The addition of ORP had a significant effect on the appearance of cooked pork batters. When increasing the added ORP content from 1% to 4%, the color of cooked pork batters became gradually darker. This was related to the color of ORP and its content when added to pork batters. Compared to the control, the addition of either 1% (T1) or 2% (T2) ORP in pork batter formulation promoted to the formation of compact and smooth appearance; however, the appearance of cooked lower-fat pork batters deteriorated as incorporation with ORP increased from 3% to 4%, exhibiting a looser and coarser appearance. These results indicated that the appropriate addition of ORP could enhance the appearance of pork batter, which corresponded to the color (Table 4) and textural properties (Table 5) of pork batter.

### 3.5. Effect of ORP on Dynamic Rheological Properties

**Figure 3:**
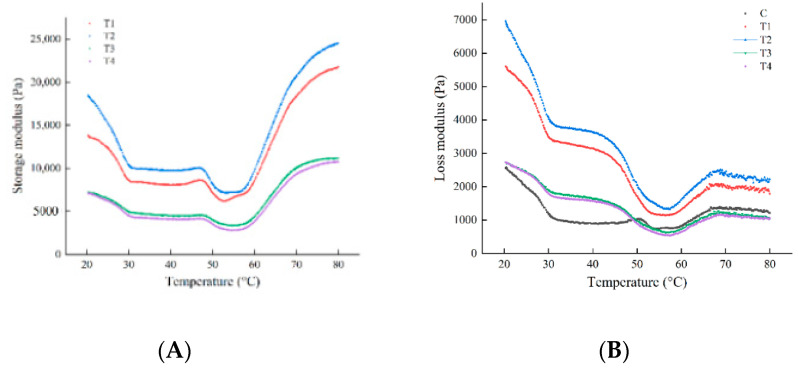
Changes in storage modulus (G′) (**A**) and loss modulus (G″) (**B**) during heating from 20 °C to 80 °C for meat batter prepared with various amounts of *Oudemansiella raphanipies* powder (ORP); C: meat batter without ORP; T1: meat batter containing 1% ORP; T2: meat batter containing 2% ORP; T3: meat batter containing 3% ORP; T4: meat batter containing 4% ORP.

The storage modulus (G′) and loss modulus (G″) changes in meat batters during heating facilitates the analysis of the ability to store and dissipate energy and the dynamic process of meat protein (mainly MP) gel formation [33]. The three-dimensional viscoelastic behavior described by the dynamic modulus reflects the denaturation and aggregation of myosin and actin during the whole heating process [34]. As observed, the higher the value of G′, the stronger the gel strength and the denser the gel structure of the meat batters [35]. The effect of ORP on the storage modulus (G′) and loss modulus (G″) of meat batter is shown in Figure 3A,B. With increasing temperature, all treatment groups exhibited the typical rheological curves of meat batters due to the thermal denaturation of muscle proteins [36]. In general, from 20 °C to 32 °C, the G′ value of meat batter presented a downward trend. G′ began to rise at approximately 41 °C, reaching a maximum value at approximately 47 °C. This phenomenon resulted from the aggregation and denaturation of heavy meromyosin, as well as the formation of an elastic gel network structure initially [37]. Afterward, G′ decreased immediately and then increased sharply at approximately 55 °C until the end phase of heating. The decrease in G′ indicated the denaturation of light meromyosin. However, the increase in G′ resulted from the formation of irreversible myosin filament compound and strengthening gel.

Compared to the controls, the G′ transition peak of all treatment groups shifted from 49.57 °C to a lower temperature 46.54 °C, possibly due to the enhanced binding of basic amino acid in ORP to the myosin head, which brought more negative charges to MP and facilitated protein unfolding and aggregation. The rheological results clearly indicated that adding 1% or 2% ORP always exhibited higher G′ compared to the control with the increasing temperature, contributing to a better ability to combine with the gel network, improving the dynamical and elastic properties of the gel. The final G′ data increased successively with increased addition level of ORP from 0 to 2%, but when the addition level of ORP increased to 3–4%, the final G′ data decreased below the control. The same tendency was observed in the final G′′ as in the final G′. These results indicated that 1–2% ORP promoted the formation of a more robust and more elastic protein gel structure. This finding coincides with the result of the textural profile of hardness (Table 5).

### 3.6. Low-Field NMR

**Figure 4:**
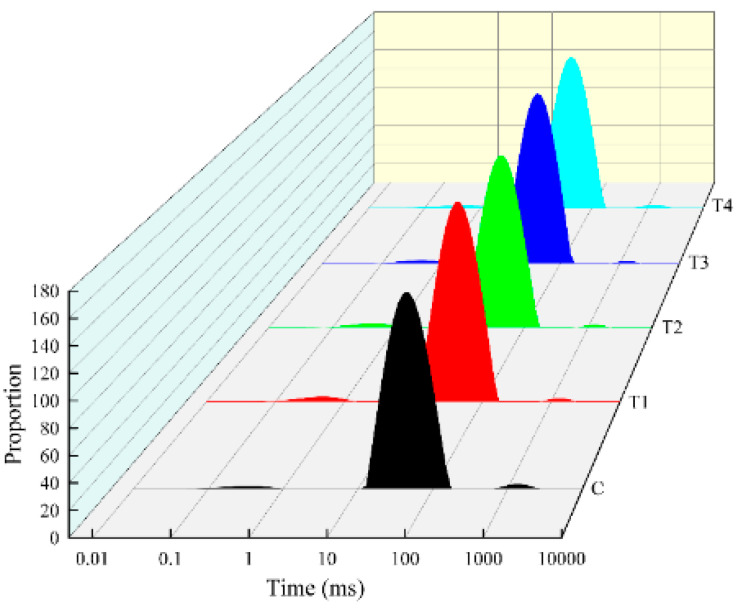
The typical distribution of nuclear magnetic resonance T_2_ relaxation times of cooked pork batters prepared with various amounts of ORP; C: meat batter without ORP; T1: meat batter containing 1% ORP; T2: meat batter containing 2% ORP; T3: meat batter containing 3% ORP; T4: meat batter containing 4% ORP.

The LF-NMR measurement of the proton transverse relaxation time T_2_ could provide critical information on the water mobility and distribution in meat gels without destroying the gel structure [38]. Three characteristic peaks (T_2b_, T_21_, T_22_) of all groups of meat batter gel corresponded to three types of water from most compactly bound to most loosely bound as shown in Figure 4. The T_2b_ (0–2 ms) reflects bound water tightly combined with macromolecules in the gel system, T_21_ (10–150 ms) reflects immobile water restricted by the protein gel structure and T_22_ (550–2400 ms) reflects free water located outside the protein gel matrix [39]. As presented in Figure 4, most of the water in the meat gel was immobile water.

The corresponding peak area fractions of three relaxation times (T_2b_, T_21_, T_22_) were expressed as P_2b_, P_21_ and P_22_, respectively. As illustrated in Table 6, no significant differences were found in the P_2b_ between the control and other treatment samples (*p* > 0.05), indicating that the distribution of bound water was not seriously influenced by the addition of ORP. The addition of 1–4% ORP induced a significant increase from 96.91 (the control) to 97.73 (*p* < 0.05), which then decreased to 97.28, with the maximum effect at 3% ORP, which could be because ORP, as a natural edible fungi with a high content of umami amino acids, probably interacts with myofibrillar proteins and binds more water molecular in the meat batter gel; thus, more immobile water was entrapped in the gel matrix. With the increase in ORP, P_22_ showed a trend of significantly decreasing and then increasing, and all treatments were significantly lower than the control group (*p* < 0.05); the mobility of water molecules in meat batter was reduced and a lower amount of free water was removed from the gel network, revealing that some free water was converted to immobile water with the addition of ORP. Overall, the moderate addition of ORP could effectively influence the water distribution of meat batter gel by improving the binding ability of immobile water and accelerate the conversion of free water into immobile water, which may explain the higher cooking loss and WHC (Figure 1) in the T1–T4 meat batter gels compared to the control.

### 3.7. Raman Spectrometry Analysis

**Figure 5:**
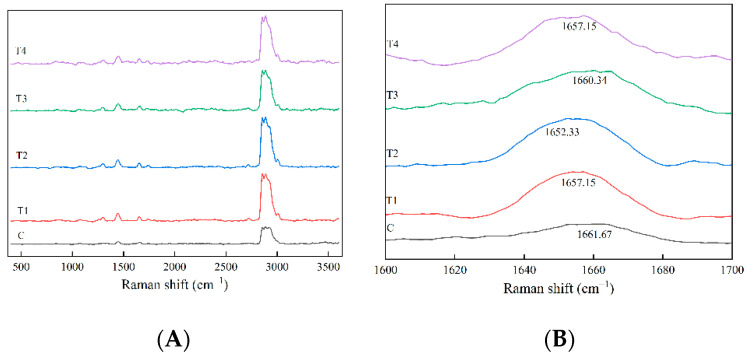
Raman spectra in the regions of 400–3600 cm^−1^ (**A**) and 1600–1700 cm^−1^ (**B**) of proteins in the cooked pork batters prepared with various amounts of ORP.

Raman spectroscopy is a valuable and non-invasive technique for providing information on the molecular conformation of meat proteins in lower-fat meat batter with various concentrations of ORP [40,41]. The secondary structure of protein is one of the most important factors affecting the quality characteristics of meat gel. The Raman spectra in the regions from 400 to 3600 cm^−1^ of proteins in the cooked pork batters prepared with various amounts of ORP are displayed in Figure 5A. The secondary structure of protein changes in the cooked pork batter were analyzed by investigating the characteristic peaks of amide I vibrational mode (1600–1700 cm^−1^) in Raman spectra (Figure 5B). The amide I band of the C group was 1661.67 cm^−1^, and for the TI–T4, it was 1657.15 cm^−1^, 1652.33 cm^−1^, 1660.34 cm^−1^ and1657.15 cm^−1^, respectively. Based on the exact location of the amide I, the secondary structure of MP was further quantitatively estimated.

As shown in Table 7, as the ORP concentration increased from 1% to 4%, the α-helix content increased from 21.96% to 46.56% *(p* < 0.05), while the T1 and T2 gels had a significantly lower α-helix content than the C gel (*p* < 0.05). Since the α-helical structure is related to the hydration capacity of MP, an increase in α-helical content may increase the hydration capacity of MP. The variation tendencies of the β-sheet, β- turn and random coil contents were opposite to those of the α-helical content; that is, their contents all decreased when the ORP was added, at the same time, the T1 and T2 gels had significantly higher β-sheet and β-turn contents than the C group gel (*p* < 0.05). This suggested that incorporating an appropriate amount of ORP promoted the conversion of α-helical structures to β-sheet, β-turn and random coil structures in the lower-fat meat batters.

The reduction in the α-helical fraction and the increase in the β-sheet fraction played a vital role in the strengthening of the three-dimensional protein gel structure, enhancing the gel network’s capacity to retain water [42,43], which supported the results regarding the cooking yield and water-holding capacity (Figure 2), texture profile analysis (Table 5) and LF-NMR (Table 6). Our study indicated that ORP addition plays a role in reducing cooking loss and improved lower-fat meat batter performance. Formulations manufactured with ORP may be used as animal fat replacement in emulsion-type meat products.

## 4. Conclusions

The results revealed that an appropriate ORP content (1–2%) significantly improved the cooking yield, WHC and improved the textural properties of the final meat product, obtaining a higher storage modulus (G’). Low-field NMR results showed that the addition of ORP significantly increased the peak area ratio of immobile water and reduced the peak area ratio of free water. From the protein secondary structure of pork batter, the addition of ORP induced the α-helical to β-sheet conversion in the lower-fat pork batter gel. In addition, pork batter with 1–2% ORP added significantly decreased pH and L* value, and increased a* value. These findings suggested the potential application of *Oudemansiella raphanipies* as a functional food ingredient in meat products.

## Figures and Tables

**Table 1:** Formulations of lower-fat meat batters prepared with *Oudemansiella raphanipies* powder at various concentrations.

Ingredient (g)	C	T1	T2	T3	T4
Pork lean meat	80	80	80	80	80
Pork back fat	20	20	20	20	20
ORP	0	1	2	3	4
NaCl	1.5	1.5	1.5	1.5	1.5
Tripolyphosphate	0.24	0.24	0.24	0.24	0.24
White pepper powder	0.16	0.16	0.16	0.16	0.16
Sugar	3.6	3.6	3.6	3.6	3.6
Ice water	30	30	30	30	30

ORP: *Oudemansiella raphanipies* powder. Pork back fat content in total weight of meat batter is approximately 15%; C: meat batter without ORP; T1: meat batter containing 1% ORP; T2: meat batter containing 2% ORP; T3: meat batter containing 3% ORP; T4: meat batter containing 4% ORP.

**Table 2:** The content and main chemical composition of ORP (%).

Chemical Composition	Moisture	Protein	Crude Fat	Carbohydrate	Ash	Crude Fiber
ORP	5.73 ± 0.03	26.92 ± 0.06	3.95 ± 0.04	28.05 ± 0.07	9.78 ± 0.04	6.21 ± 0.05

**Table 3:** The composition and content of hydrolyzed amino acids.

Amino Acid Species	Content (mg/g)	The Proportion (%)
Aspartic acid	6.206 ± 0.03	7.74
Threonine	3.387 ± 0.04	4.23
Serine	3.63 ± 0.03	4.53
Glutamic acid	8.326 ± 0.05	10.39
Proline	1.55 ± 0.02	1.93
Glycine	3.27 ± 0.04	4.08
Alanine	3.905 ± 0.04	4.87
Cystine	0.147 ± 0.01	0.18
Valine	7.211 ± 0.05	9.00
Methionine	2.226 ± 0.02	2.78
Isoleucine	3.659 ± 0.05	4.56
Leucine	7.779 ± 0.04	9.70
Tyrosine	2.225 ± 0.03	2.78
Phenylalanine	13.374 ± 0.05	16.68
Histidine	1.463 ± 0.01	1.83
Lysine	3.874 ± 0.01	4.83
Arginine	7.926 ± 0.02	9.89
Total	80.158 ± 0.03	100

**Table 4:** pH value and lightness (L*), redness (a*) and yellowness (b*) values of cooked lower-fat meat batter prepared with various amounts of ORP.

Samples	pH	L*	a*	b*
C	6.04 ± 0.01 ^c^	75.33 ± 0.27 ^e^	0.07 ± 0.06 ^a^	14.56 ± 0.21 ^a^
T1	6.02 ± 0.01 ^b^	68.15 ± 0.55 ^d^	2.14 ± 0.10 ^b^	14.44 ± 0.45 ^a^
T2	6.02 ± 0.01 ^b^	64.14 ± 0.28 ^c^	2.90 ± 0.10 ^c^	14.98 ± 0.50 ^a^
T3	6.01 ± 0.02 ^b^	61.53 ± 0.75 ^b^	3.07 ± 0.06 ^d^	14.82 ± 0.45 ^a^
T4	5.97 ± 0.01 ^a^	58.27 ± 0.74 ^a^	3.29 ± 0.07 ^e^	15.05 ± 0.32 ^a^

C: meat batter without ORP; T1: meat batter containing 1% ORP; T2: meat batter containing 2% ORP; T3: meat batter containing 3% ORP; T4: meat batter containing 4% ORP; different letters (a, b, c, d, e) in the same column indicate significant differences (*p* < 0.05).

**Table 5:** Textural properties of cooked lower-fat meat batters prepared with various amounts of ORP.

Samples	Hardness (g)	Springiness (mm)	Cohesiveness	Chewiness (g.mm)
C	4527.73 ± 295.67 ^c^	0.82 ± 0.02 ^ab^	0.701 ± 0.004 ^c^	2702.37 ± 198.29 ^b^
T1	5688.86 ± 171.52 ^e^	0.85 ± 0.01 ^bc^	0.699 ± 0.003 ^c^	3388.51 ± 97.05 ^c^
T2	5240.44 ± 201.43 ^d^	0.86 ± 0.04 ^c^	0.606 ± 0.003 ^b^	2790.72 ± 165.57 ^b^
T3	4199.16 ± 176.81 ^b^	0.80 ± 0.02 ^a^	0.475 ± 0.002 ^a^	1634.73 ± 180.72 ^a^
T4	3879.20 ± 49.64 ^a^	0.80 ± 0.01 ^a^	0.473 ± 0.004 ^a^	1493.88 ± 91.28 ^a^

C: meat batter without ORP; T1: meat batter containing 1% ORP; T2: meat batter containing 2% ORP; T3: meat batter containing 3% ORP; T4: meat batter containing 4% ORP; different letters (a, b, c, d, e) in the same column indicate significant differences (*p* < 0.05).

**Table 6:** T_2_ relaxation corresponding peak area proportion of cooked pork batters prepared with various amounts of ORP.

Samples	C	T1	T2	T3	T4
P_2b_	0.71 ± 0.02 ^ab^	0.55 ± 0.05 ^a^	0.86 ± 0.02 ^b^	1.09 ± 0.21 ^b^	0.66 ± 0.02 ^a^
P_21_	96.91 ± 0.21 ^a^	97.51 ± 0.04 ^c^	97.70 ± 0.03 ^cd^	97.73 ± 0.08 ^d^	97.28 ± 0.08 ^b^
P_22_	2.38 ± 0.21 ^d^	1.95 ± 0.04 ^c^	1.44 ± 0.02 ^b^	1.17 ± 0.04 ^a^	2.06 ± 0.05 ^c^

C: meat batter without ORP; T1: meat batter containing 1% ORP; T2: meat batter containing 2% ORP; T3: meat batter containing 3% ORP; T4: meat batter containing 4% ORP; different letters (a, b, c, d) in the same column indicate significant differences (*p* < 0.05).

**Table 7:** Relative content of the secondary structure of proteins in the cooked pork batters prepared with various amounts of ORP.

Samples	α-Helix (%)	β-Sheet (%)	β-Turn (%)	Random Coil (%)
C	37.67 ± 0.59 ^c^	24.49 ± 0.07 ^b^	14.96 ± 0.10 ^b^	22.89 ± 0.52 ^c^
T1	21.96 ± 0.63 ^a^	36.56 ± 1.11 ^d^	16.79 ± 0.46 ^d^	24.69 ± 0.20 ^d^
T2	33.84 ± 0.07 ^b^	27.50 ± 0.11 ^c^	15.70 ± 0.60 ^c^	22.96 ± 0.70 ^c^
T3	42.15 ± 0.62 ^d^	23.71 ± 0.38 ^b^	13.94 ± 0.46 ^a^	20.20 ± 0.20 ^b^
T4	46.56 ± 0.52 ^e^	22.03 ± 0.10 ^a^	13.43 ± 0.12 ^a^	17.98 ± 0.52 ^a^

C: meat batter without ORP; T1: meat batter containing 1% ORP; T2: meat batter containing 2% ORP; T3: meat batter containing 3% ORP; T4: meat batter containing 4% ORP; different letters (a, b, c, d, e) in the same column indicate significant differences (*p* < 0.05).

## Data Availability

The data presented in this study are available on request from the corresponding author.
